# Portable X-Ray Fluorescence Spectroscopy for Rapid and Cost-Effective Determination of Elemental Composition of Ground Forage

**DOI:** 10.3389/fpls.2019.00317

**Published:** 2019-03-19

**Authors:** Yadav Sapkota, Louis M. McDonald, Thomas C. Griggs, Thomas J. Basden, Brandon Lee Drake

**Affiliations:** ^1^Division of Plant and Soil Sciences, West Virginia University, Morgantown, WV, United States; ^2^Wetland and Aquatic Biogeochemistry Laboratory, College of the Coast and Environment, Louisiana State University, Baton Rouge, LA, United States; ^3^Extension Service, West Virginia University, Morgantown, WV, United States; ^4^Department of Anthropology, The University of New Mexico, Albuquerque, NM, United States

**Keywords:** PXRF, forage, analysis, particle size, scan time

## Abstract

The recent development of portable X-ray fluorescence spectrometers (PXRF) has created new avenues for rapid plant elemental concentration determination at reduced cost while avoiding hazardous chemicals. A few studies have indicated the potential use of PXRF for homogenous plant tissue analysis. However, there is a lack of information for analysis of heterogeneous plant samples like livestock forage, which consists of a mixture of several species and plant parts, each varying in elemental concentration. Our objective was to evaluate PXRF for forage analysis, specifically the effect of forage particle size and scan time on important elements including P, K, Ca, and Fe determination. Hay samples (*n* = 42) were oven dried (60°C for 3 days) and ground into three particle sizes (≤0.5 mm, 0.25–0.5 mm and 1–2 mm). Prepared samples were scanned by PXRF using a vacuum (<10 torr) without a filter. Samples were placed in cups over thin prolene X-ray film and scanned for 180 s. A subset (*n* = 29) were also scanned for 60 and 120 s. PXRF counts for P, K, Ca, and Fe were compared with laboratory Inductively Coupled Plasma Optical Emission Spectroscopy (ICP) determinations, using regression models. Results indicated that these elements could potentially be determined with PXRF (*r*^2^ ≥ 0.70) in heterogeneous forage samples. Relationship strength increased with decreasing particle size, however, the relationship was still strong (*r*^2^ ≥ 0.57) at the largest particle size. Scanning time did not affect the relationship with ICP concentration for any of the particle sizes evaluated. This work demonstrated that with the right sample preparation PXRF can obtain results comparable to acid digestion and ICP regardless of sample composition, and suggests the potential for *in situ* determinations.

## Introduction

Forage is a major ruminant livestock feed and source of minerals including P, K, Ca, Mg, and Fe. Animal performance and health are directly influenced by the mineral content of forages (Minson, 1990; Van Soest, 1994). Deficiencies or excesses of specific mineral elements in forage decrease animal performance and economic return (Reid et al., 1970). The quantity of forage essential to each animal type is determined by the expected level of animal performance, forage quality, and mineral content. Large herds require enormous amounts of feed and forage for daily consumption, but the nutritional status of the forage changes over time, indicating the need for regular sampling and analysis for a balanced ration; increasing financial burden to farmers (Berzaghi et al., 2005). Use of near-infrared reflectance spectroscopy is commonly used for forage quality analysis. However, minerals are often still quantified with expensive and time-consuming wet-chemical methods; acid digestion followed by atomic spectroscopic techniques (Karla, 1998). Although these provide accurate quantification of minerals, they can have serious shortcomings. They require destructive sampling, and are costly, time-consuming, and generate hazardous waste. The typical cost of forage analysis (minerals only) in the Mid-Atlantic region is $28 per sample (e.g., Cumberland Valley Analytical Services, 2018). Assuming that half of the 20,600 livestock farms in West Virginia (West Virginia Department of Agriculture, 2017) submits 4–8 forage samples annually, the annual cost of forage analysis for mineral determination is 1.2–2.3 million dollars; significantly more nationally. Along with this cost, the generation of hazardous waste is a consideration. X-ray fluorescence (XRF) could overcome many of these disadvantages of wet chemical analysis and allows quick determination of elemental concentrations at reduced cost in plant samples (Reidinger et al., 2012; Towett et al., 2016; McGladdery et al., 2018).

X-ray photons are emitted from an X-ray tube by the interaction of electrons with a metal anode. When the energy of the incident X-ray is greater than the binding energy of electrons in the shell, inner electrons are ejected, leaving a vacant space. In order to fill this vacant space, the electron from a higher shell moves to the inner shell, emitting secondary X-ray radiation of energy/wavelength characteristic to each element (Bruker, 2016). The emitted radiation is then detected. Portable XRF (PXRF) is based on an energy dispersive principle in which dispersion of the entire spectrum occurs directly in the detector in the energy domain (Piorek, 1997). X-ray energy is inversely proportional to wavelength and is expressed as keV (kilo-electron volt) (Kalnicky and Singhvi, 2001). When a sample is scanned, the resulting spectrum identifies the element (peak position or energy); the area under the peak (intensity) is proportional to the concentration of the elements present in the sample (Willis and Duncan, 2008; Weindorf et al., 2014). PXRF may be qualitative, semi-quantitative, or quantitative as an analytical technique. Quantitative data are obtained by calibrating the PXRF with reference wet chemical methods (Maarschalkerweerd and Husted, 2015) or standard addition methods (Reidinger et al., 2012). Some portable units are comparable to benchtop XRF in elemental quantification and limits of detection (Bueno Guerra et al., 2014) but superior in terms of portability, cost-effectiveness, simplicity of operation, potential for *in situ* measurement, and analysis of large samples (Analytical Methods Committee et al., 2008; Bueno Guerra et al., 2014). In addition, it can be a superior alternative to wet chemistry in terms of cost- and time-effectiveness, and non-destructive analysis of samples (Reidinger et al., 2012).

Previous studies on plant elemental compositional analysis have indicated some avenues to analyze forage samples by PXRF. Bueno Guerra et al. (2014) found a good correlation (*r* = 0.91–0.99) between acid digestion values and PXRF counts for P, K, Ca, P, K, Ca, S, Fe, Mn, and Si using the top visible dewlap leaves of 23 sugarcane varieties. Reidinger et al. (2012), obtained a linear calibration curve for silicon in Si-spiked methylcellulose between acid digestion and portable XRF counts. Likewise, they found a good correlation (*r* = 0.98) for P determination in certified plant reference material. Towett et al. (2016), using diverse plant samples, found that direct contact on the surface of a PXRF with the aid of vacuum provided highest sensitivity and accuracy (*r*^2^ > 0.90) for light elements (Mg to P) compared to using prolene sample cups. However, compromising some lower detection limits, elements like S, K, and Ca could be analyzed without vacuum. The use of sample cups negatively affected the measurement of elements, indicating the potential for the *in situ* analysis of plant samples. Kalcsits (2016) found significant correlations (*r* = 0.73–0.97) between PXRF measurement and wet chemical analysis for Ca and K concentrations in apple and pear fruits. McLaren et al. (2012) used PXRF to evaluate the effect of scanning time and particle size on data quality using four plant species: corn tops (2 mm), wheat tops (2 mm and powder), cotton leaves (powder), and soybean grains (powder). They found a significant linear relationship between the acid digest and portable XRF readings for Ca, Co, Cr, Fe, K, Mn, Ni, P, S, Si, and Zn in three plant species (corn, cotton, and soybean). Likewise, they found similar *r*^2^ values for the same sample at different scan times. Zinc and Fe concentrations by PXRF in rice and pearl millet grain (Paltridge et al., 2012a) were highly correlated with Inductively Coupled Plasma Optical Emission Spectroscopy (ICP) values (*r*^2^ = 0.79–0.98). PXRF predicted Zn and Fe in rice within 1.9 and 1.6 mg kg^-1^ of ICP values, and in pearl millet within 7.6 and 12.5 mg kg^-1^ of ICP values at a 95% confidence level. In a similar experiment with whole wheat grain, Paltridge et al. (2012b) found PXRF values for Zn, Fe, and Se were highly correlated with ICP values. Standard errors of prediction were 2.2, 2.6, and 1.5 mg kg^-1^ for Zn, Fe, and Se, respectively. Likewise, McGladdery et al. (2018), using thatch, deciduous leaves, single species of grasses, tree bark and herbaceous plants, demonstrated that PXRF is a useful approach for elemental determination (Zn, Pb, Cd, and Fe) in vegetation samples.

Accurate determination of composition depends on proper sample preparation, sample introduction, instrumental setup of the XRF (Towett et al., 2016), the energy level of the element, scanning time, particle size and moisture content of the sample (McLaren et al., 2012). In addition, enhancement effects and penetration and escape depths are potential influential factors in PXRF analyses (Bruker, 2016). Penetration depth refers to the depth to which the primary X-ray penetrates the sample and depends on the sample matrix and the primary X-Ray energy. Escape depth also depends on the sample matrix but now it’s the emergent, fluorescent X-ray energy that is relevant. Although PXRF can measure most elements, air attenuation of low energy X-rays restricts the measurement of light elements, especially those below Si (Potts and West, 2008), except when equipped with a unique chamber capable of working under vacuum, or a helium atmosphere (Brouwer, 2013; Bueno Guerra et al., 2014). A vacuum or helium is used to remove the interfering air between the instrument window, on which the sample rests and the detector. The elements from Mg to Fe in periodic table are considered light element while the elements >Fe are considered heavy elements in PXRF analysis. The primary fluorescence produced by higher energy elements in a composite sample causes enhancement of the lower energy elements through the emission of secondary fluorescence (Salesh, 1988). The fluorescence that reaches the detector is the combination of primary and secondary fluorescence. It is possible to have tertiary or even higher-level fluorescence, however, it is almost negligible in practice (Brouwer, 2013). Some PXRF units have the ability to control energy, current, and use of filters for the determination of a specific range of elements. There is a general rule that to get fluorescence from elements, at least 2 KeV more energy should be applied to the sample (Bruker, 2016). For the detection of the light elements (Mg to Fe), a voltage level of 15 KeV and anode current of 26 μA is used. The greatest benefit associated with this setting is the reduction of enhancement effects due to the fluorescence associated with higher energy elements. The intensity of radiation increases with sample thickness up to a point called critical thickness beyond which thickness is assumed to be infinity (Sitko, 2009). The critical penetration and escape depths are the thickness of the sample beyond which almost all of the emitted x-rays are absorbed, which is very low (in μm range) for light elements (Potts and West, 2008). The estimated analytical measurement depth of P is 80 μm in water, 70 μm in cellulose, and 60 μm in fructose (Towett et al., 2016); P is measured almost at the surface of the sample reducing the influence of sample thickness. The measurement depth increases with increase in the atomic number of the element.

The sample surface texture is also important in XRF measurements. Ideally the surface of the sample should have mirror finish, which can only be realized with fine powders, fused beads, and metals (Willis and Duncan, 2008). PXRF intensity increases with a decrease in sample particle size. The particle size effect is more pronounced in light elements like P, K, Ca, and Fe than heavy elements because of low energy level and lower penetration depth (Maruyama et al., 2008). In addition, if the sample is of heterogeneous particle size, incoming X-rays will not irradiate all particles and thus no fluorescence will be received from shadowed particles (Yamada, 2014). The best approach to deal with a sample particle size is to grind to size less than the measurement depth of the elements to be determined (Willis and Duncan, 2008). However, grinding samples to fine power is not always beneficial; there is the possibility of contamination by blades in grinding mills. Imanishi et al. (2010) in a study with soil samples found that particle size of soil samples affected XRF intensity in the determination of light elements. Samples with small particles produced better results for light elements than large particle sizes.

Raw peak count and spectral resolution increase with increasing scan time (Kalnicky and Singhvi, 2001; McLaren et al., 2012). Scanning samples for longer time improves detection limit, however, the number of samples analyzed will be reduced (Kalnicky and Singhvi, 2001) and sample radiation damage is possible. Bueno Guerra et al. (2014) optimized measurement time as 150 s by scanning a pellet of NIST SRM 1515 apple leaves from 10 to 300 s. The coefficient of variation (CV) ranged from 0.1% for Ca to 3.3% for P at 150 s. Reidinger et al. (2012) used 30 s for scanning pelletized ground certified reference material of different plant species and *Deschampsia caespitosa* leaves. Towett et al. (2016) used 180 s for diverse fine powdered (<53 μm) plant samples. Paltridge et al. (2012a) used 60 s for the determination of Zn and Fe in rice and pearl millet and for the determination of Zn, Fe, and Se in whole grain wheat (Paltridge et al., 2012b). McLaren et al. (2012) evaluated scan time (120 and 300 s) for cotton leaf powder. The spectral peaks were higher for 300 s than 120 s scanning time in cotton powder. Even though the two scan times produced similar data quality, regression slopes will be greater for longer scanning time thus increasing the accuracy of measurement (Kalnicky and Singhvi, 2001; McLaren et al., 2012).

The few studies presented above indicate the potential of PXRF analysis in plant sample analysis, at least for specific plant parts, mostly leaves and seeds. We are not aware of studies evaluating PXRF for heterogeneous plant material like forage (grasses, legumes, and mixtures), despite the importance of forage for ruminant animal agriculture. A forage sample may contain all parts of the plant (grasses, legumes, and forbs; stems, seeds, and leaves), each with potentially variable mineral content. Here we assumed, due to hetegenous nature of the forage samples and smooth surface and homogenity requirement of the PXRF analysis, that sample particle size would be the most limiting factor for forage analysis. The overall goal of this paper was to provide theoretical and practical information for the rapid and cost-effective determination of light elements (≤Fe) in heterogeneous forage samples using PXRF.

## Materials and Methods

### Sample Collection and Preparation

**FIGURE 1:**
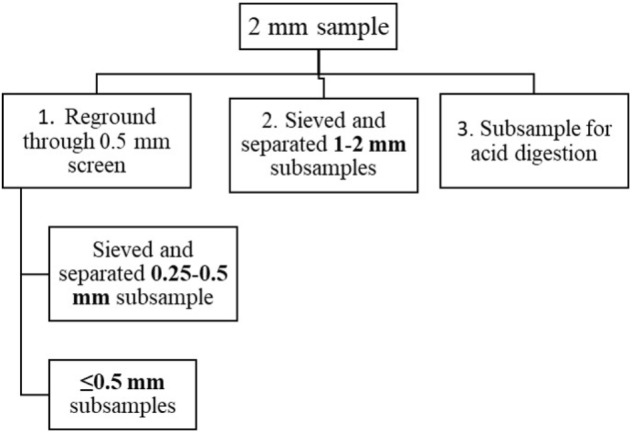
Schematic of subsampling of 2 mm samples into different particle sizes. 2 mm particle sized samples were obtained by grinding dried samples and allowed to pass through 2 mm screen of a cyclone mill.

Samples (*n* = 42) were collected from hay bales from different West Virginia farms using a plunge corer in summer and fall 2016. Samples were cored from hay bales representing legumes, grass-legume mixture, and mixed grasses having first and regrowth cuttings. Collected samples were placed in paper bags and dried in an oven at 60°C for 72 h. Milling of dried samples was done in a cyclone mill (FOSS Tecator 1093, FOSS North America, Eden Prairie, MN, United States). The whole sample was allowed to pass through a 2 mm screen and was split into three parts (Figure 1) using the alternate shoveling method (Thiex et al., 2002). One part was re-ground through a 0.5 mm screen and split into two parts. One part (hereafter called as ≤0.5 mm samples) was used for evaluating the suitability of PXRF for forage P, K, Ca, and Fe determination.

In order to analyze the particle size of samples, a sieve was used to separate the other particle sizes (Figure 1); 1–2 mm size (particles that pass through 2 mm but retained at 1 mm mesh) were prepared from a part of 2 mm particles. Likewise, 0.25–0.5 mm size (particles that pass through 0.5 mm but retained at 0.25 mm mesh) were prepared from a part of ≤0.5 mm samples. Thus, each hay sample had three particle size subsamples ≤0.5 mm, 0.25–0.5 mm, and 1–2 mm.

### Wet Chemical Analysis

Subsamples (from a ground sample that passed through 2 mm screen) of each sample were acid-digested in triplicate by microwave (MARS Xpress, CEM Inc., Matthews, NC, United States) and the elemental concentration was determined using ICP (Optima DV 2100, PerkinElmer, Norwalk, CT, United States). A NIST Certified Reference Material (CRM), 1573a-tomato leaf, was digested with samples to check the accuracy of digestion. Exactly 10.0 mL 70% nitric acid was added to 0.50 g sample in digestion vessels and placed in a fume hood for 1 h to eliminate gasses produced. The tubes were placed in a microwave, heated for 15 min at 200°C followed by holding at 200°C for 15 min and allowed to cool overnight. The digested liquid was transferred into test tubes, diluted and brought to a final volume of 50 mL. The diluted liquid was filtered before analysis by ICP. The elemental concentration determined by wet chemical analysis are abbreviated as ICP values.

### PXRF Assays

Samples were scanned using portable XRF (Tracer III-SD; SN T3S2102; Bruker Elemental, Kennewick, WA, United States) in benchtop mode equipped with a 4W rhodium tube, and a Peltier-cooled, 10 mm^2^ silicon drift detector, with a voltage of 15 KeV and anode current of 26 μA without a filter. It was operated using a computer and internal vacuum (<10 torrs) to reduce air attenuation. Vacuum is an optional external attachment that comes with PXRF unit. Vacuum is connected to PXRF unit through a tube which reduce the X-ray attenuation by atmospheric air inside the unit. Samples were placed in double open-ended sample cups (Chemplex Industries Inc., United States) over a thin X-ray film (4 μm prolene) in order to homogenize the depth of the sample used and, also, to preserve samples for future use. The cups containing ground samples were placed on the nose of the PXRF and covered with an X-ray shielding lid. All samples of three particle sizes (≤0.5 mm, 1–2 mm, 0.25–0.5 mm) were scanned twice for 180 s. Some samples (*n* = 29) of 0.25–0.5 mm and 1–2 mm particle size were also scanned for the 60 and 120 s for scan time analysis.

### Data Collection and Statistical Analysis

The spectrum of each analysis and count per second (intensity in cps) was collected using S1PXRF software (Bruker Elemental, Kennewick, WA, United States). Data were organized in Microsoft Excel and analyzed by JMP (Version Pro 12.2, SAS Institute Inc., Cary, NC, United States), and R (R Foundation for Statistical Computing, Vienna, Austria). The coefficient of variation (CV) for triplicates of ICP determined values of samples and CRM were calculated from Microsoft Excel. The summary statistics of ICP determined values and PXRF intensity were obtained from JMP. The PXRF counts between two particle sizes (0.25–0.5 and 1–2 mm) were compared using Welch two sample *t*-test in R. The intensity of P, K, Ca, and Fe of all particle sizes and scan times were compared with ICP determined concentrations using simple linear regression model in R. In R, *lm()* function was used for simple linear regression and regression parameters were obtained using *summary()* function. The regression plots were prepared using *qplot()* function of the package ggplot2 (Wickham, 2016) in R. Significance criterion (alpha) for all tests was 0.05.

## Results

### Wet Chemical Elemental Concentrations

The CVs of triplicate wet chemical analyses of the samples were 3.9% for P, 6.3% for K, 1.5% for Ca and 3.4% for Fe. The CVs for CRM-tomato leaf digestion were 5.5% for P, and 3.9% for K, 4.2% for Ca, and 8.3% for Fe (Table 1). A CV below 10% is acceptable for elemental determination in plant samples (Bueno Guerra et al., 2014). Likewise, the difference between the standard value from National Institute of Standards and Technology (NIST) and ICP-determined value of CRM, 4.1% for P, 4.8% for K, 2.4% for Ca and 13.6% for Fe (Table 1), were within the acceptable range (Bueno Guerra et al., 2014).

**Table 1:** Coefficient of variation (CV) for triplicates of forage sample digestion (ICP concentrations) (*n* = 42) and CV for CRM digestion.

Element	CV for forage samples (%)	CV for CRM (%)	Difference between standard and ICP concentration for CRM (%)
P	4.9	5.5	4.09
K	6.4	3.9	4.80
Ca	1.5	4.2	2.42
Fe	3.4	8.3	13.64


The CVs for elemental concentrations between forage samples (ICP values) were in relatively wide ranges of P-28%, K- 23%, Ca- 32%, and Fe- 52% (Table 2), indicating that a diverse set of forage samples had been obtained.

**Table 2:** Summary statistics for ICP determined elemental concentrations (*n* = 42).

Statistic	Elemental concentration (mg/kg)			
	P	K	Ca	Fe
Mean	2,100	17,100	4,700	200
Minimum	1,000	11,500	2,700	44
Maximum	3,500	27,400	9,000	448
*SD*	600	4,000	1,500	105
CV (%)	28	23	32	52


**FIGURE 2:**
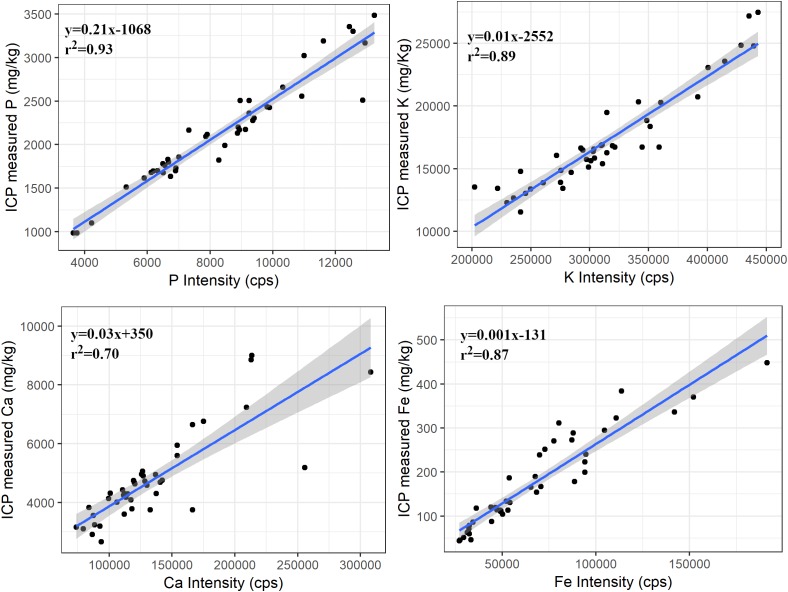
Regression plots between ICP measured concentrations and PXRF intensity of P, K, Ca, and Fe for the ≤0.5 mm samples. The shaded portion represents the standard error of the regression line. The relationship was significant (*p* < 0.001) for all elements.

Suitability of PXRF in forage analysis was determined by comparing PXRF counts (of ≤0.5 mm samples) obtained by scanning for 180 s with the ICP-determined concentration. Regression models between ICP determined concentration and PXRF counts were significant (*p* < 0.001) for P, K, Ca, and Fe. These elements had coefficient of determination (*r*^2^) ranging from 0.70 for Ca to 0.93 for P (Figure 2), indicating a strong relationship between PXRF measured intensity and ICP measured concentration.

### Effect of Sample Particle Size

**FIGURE 3:**
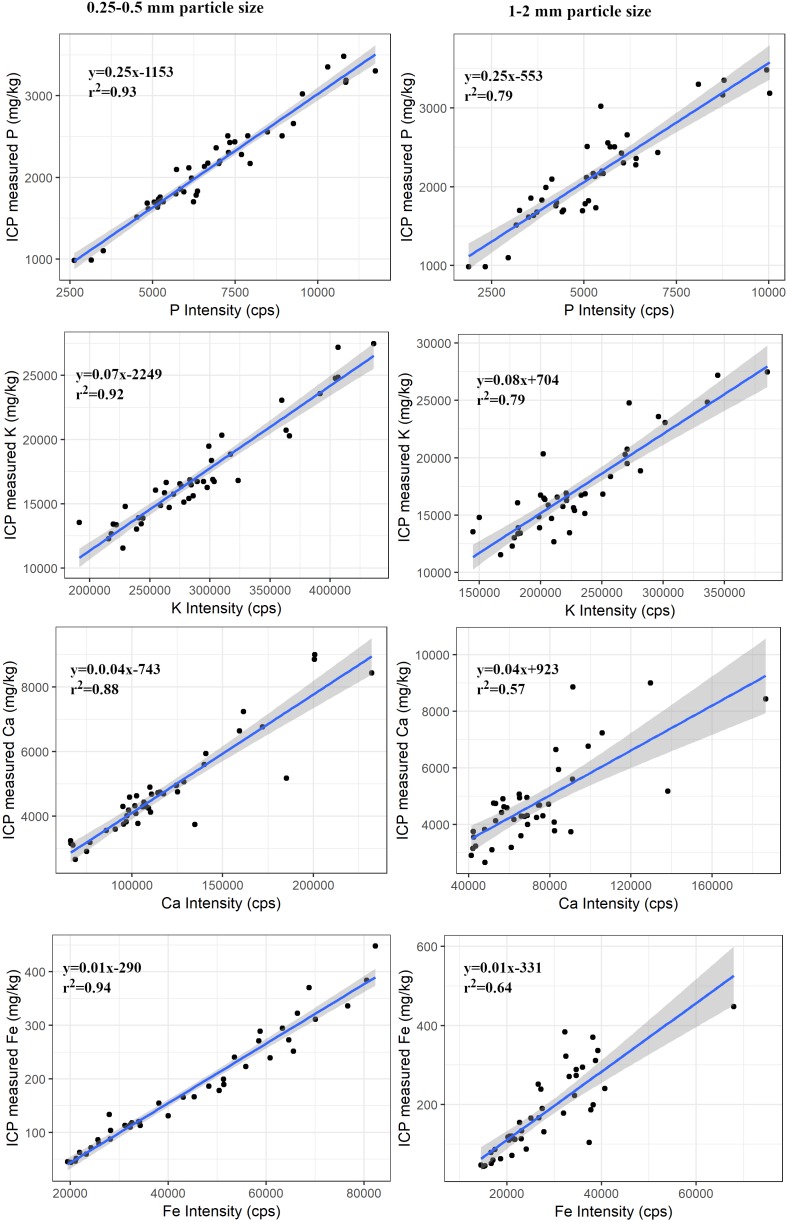
Regression plots between ICP measured concentration and PXRF intensity for P, K, Ca, and Fe for two particle size of forage samples. The shaded portion shows the standard error. The relationship was significant (*p* < 0.001) for all elements in the samples of both particle sizes.

There were significant differences (*p* < 0.001) in the intensity between two particle sizes (0.25–0.5 mm and 1–2 mm) of the samples for each element. There was a decreasing trend in the photon counts with an increase in particle size of the samples (Table 3). However, regression models between PXRF counts and ICP determined concentration were significant (*P* < 0.001) for P, K, Ca, and Fe in the samples of both particle size. There was a decreasing trend in *r*^2^ values with increase in the particle size of the sample. The decrease in *r*^2^ values was 0.14 for P, 0.13 for K, 0.31 for Ca, and 0.30 for Fe (Figure 3), indicating the influence of particle size of the sample in elemental concentration determination.

**Table 3:** Summary statistics for PXRF intensities (cps) for three different particle size forage samples scanned for 180 s (*n* = 42).

Particle size	Statistic	PXRF intensities (cps)			
		P	K	Ca	Fe
PXRF (≤0.5 mm)	Mean	15,700	2,84,900	1,46,800	98,800
	Minimum	10,300	1,88,700	1,00,500	63,600
	Maximum	21,900	3,99,300	2,94,700	2,05,300
	*SD*	2,900	54,200	41,900	31,500
	CV (%)	18	19	28	32
PXRF (0.25–0.5 mm)	Mean	13,400	2,64,200	1,30,800	77,100
	Minimum	8,900	1,78,200	87,400	54,900
	Maximum	19,100	3,93,500	2,26,700	1,11,400
	*SD*	2,400	51,700	33,600	16,800
	CV (%)	18	19	26	22
PXRF (1–2 mm)	Mean	10,800	2,08,800	86,500	58,000
	Minimum	7,000	1,35,800	56,200	43,900
	Maximum	16,700	3,45,600	1,85,300	95,000
	*SD*	2,200	44,800	25,600	9,600
	CV (%)	20	21	30	16


### Effect of Scan Time

Net counts per second increased with increasing scan time. There were significant (*p* < 0.001) relationships between PXRF counts at three different scan times (60, 120, and 180 s) and ICP-determined concentration of all elements in samples of both particle size (0.25–0.5 mm and 1–2 mm). Scanning for longer time did not increase r^2^ values for the samples of either particle sizes (Table 4). For example, *r*^2^ values for P measured from 0.25–0.5 mm particle size samples were 0.88, 0.84, and 0.88 for three scan time (60, 120, and 180 s), respectively. These significant relationships (*p* < 0.001) with the ICP determined concentrations along with similar *r*^2^ values indicate the nominal effect of the scan time on elemental quantification and are irrespective of the particle size. However, increasing scanning time increased net counts and thus the slope of the regression line decreased (Table 4).

**Table 4:** Coefficient of determination (*r*^2^), Root mean square error (RMSE), slope, and *p*-value of regression between ICP determined values (mg/kg) with the PXRF intensity (cps) at 3 scan times (60, 120, and 180 s) for samples of two particle sizes (0.25–0.5 mm and 1–2 mm) (*n* = 29).

Element	Scan time (s)	Regression of ICP values with PXRF intensity at different scan time for 0.25–0.5 mm samples					Regression of ICP values with PXRF intensity at different scan time for 1–2 mm samples				
		*r*^2^	RMSE	Intercept	Slope	*p*-value	*r*^2^	RMSE	Intercept	Slope	*p*-value
P	60	0.88	212	–1,112	0.72	<0.001	0.74	314	–276	0.68	<0.001
	120	0.84	236	–186	0.27	<0.001	0.74	307	–344	0.35	<0.001
	180	0.88	210	–1,156	0.25	<0.001	0.72	316	–413	0.24	<0.001
											
K	60	0.85	1,763	–4,751	0.23	<0.001	0.77	2,160	–2,168	0.26	<0.001
	120	0.87	1,664	–4,447	0.11	<0.001	0.78	2,171	–2,148	0.13	<0.001
	180	0.87	1,647	–5,131	0.08	<0.001	0.80	2,040	–2,283	0.09	<0.001
											
Ca	60	0.97	300	–1,165	0.14	<0.001	0.70	941	416	0.15	<0.001
	120	0.95	372	–823	0.06	<0.001	0.69	964	430	0.08	<0.001
	180	0.97	303	–1,132	0.05	<0.001	0.72	917	200	0.05	<0.001
											
Fe	60	0.93	22	–297	0.02	<0.001	0.42	65	–238	0.02	<0.001
	120	0.88	30	–297	0.01	<0.001	0.41	65	–232	0.01	<0.001
	180	0.94	22	–292	0.01	<0.001	0.59	55	–326	0.01	<0.001


## Discussion

Portable X-ray fluorescence measurements had a strong relationship with the elemental concentrations from wet chemical methods. Despite the complex matrix of forage samples, PXRF was able to predict elemental concentration accurately. The particle size of the sample affected the result. The net counts per second were decreased with increase in particle size and the strength of the relationship with ICP values also decreased. The result is consistent with the theory of XRF (Maruyama et al., 2008). This is mainly due to the scattering of X-rays by uneven surfaces and shadowing effects (Yamada, 2014); light elements like P, K, Ca, and Fe are more susceptible to this effect due to their low energy and shallow penetration depth. The best solution to minimize particle size effect is to grind samples to the size less than their measurement depth (Willis and Duncan, 2008) but grinding to finer particle size is not always feasible logistically and presents a potential risk of contamination. There are tradeoffs between the desired accuracy and feasibility of sample preparation. From our study we can say that grinding samples to below 0.25 mm provided no benefit relative to the sample sizes 0.25–2 mm. In most cases grinding samples to pass through 2 mm screen should be acceptable. This also indicate that heterogeneous nature of the forage samples does not have significant effect on the results. The PXRF was able to predict the elemental concentration from the mixture of particles varying in elemental concentration. The acceptable results for the larger particles size of the samples also indicate the potential for *in situ* measurements.

Scanning sample for a longer period of time did not produce a significant effect on elemental quantification of the samples of either particle size. The increase in photon counts and spectral resolution increasing scan time was consistent with the literature (Kalnicky and Singhvi, 2001; McLaren et al., 2012). Though scanning samples for longer time improved detection limits (Kalnicky and Singhvi, 2001), the strength of the relationship with ICP values was not affected. The increase in the photon counts changed the regression slopes, thus improving detection limit (Kalnicky and Singhvi, 2001; McLaren et al., 2012). Scanning for longer time decreases the number of samples analyzed and radiation damage to sample is possible. Our results indicate that forage samples can be analyzed with as little as 60 s without losing accuracy. This further strengthens the rapidity of the PXRF measurements. However, if we are interested in elements present in lower concentrations, longer scan times might be necessary.

This method of forage analysis is cost-effective and rapid. Once a sample is prepared (dried and ground) it takes just minutes to get PXRF results, compared to hours with wet chemical digestion. Also, the initial investment is less than that needed for wet chemistry equipment. Other than replacing windows, there are no other operational costs (except the operator) associated with PXRF and analysis is quite easy and straightforward. Simple training on the instrument is sufficient to process the samples. However, the wet chemistry has a higher operational cost associated with instruments (ICP, microwave digestion), chemicals, and other supplies.

Along with sample preparation, sample introduction and instrumental setup of the XRF (Towett et al., 2016), air attenuation, enhancement effects, escape and penetration depth and thickness of the sample affect XRF results (Brouwer, 2013). The custom setting and accessories associated with PXRF has potential to remove, adjust or minimize these problems. Presence of vacuum facility removes air attenuation problem associated with the determination of light elements like P, K, and Ca. The use of different factory settings of the X-ray tube is useful in removing the effects like enhancement effect. For the detection of light elements, low voltage (15 Kev) is sent which only excite up to Fe in the periodic table. There are very low chances of enhancing the fluorescence of light elements by heavier elements. Likewise, light elements like P, K, and Ca have low measurement depth which helps to minimize of the effect of thickness of the sample.

## Conclusion

Mineral composition data in forage is an important part of the animal feeding plan. Since PXRF results were in close agreement with ICP results, it can be used as a prominent technique in elemental determination in forage samples and potentially other mixed animal feeds. It removes several disadvantages of traditional wet chemical analysis techniques and provides elemental concentration quickly at a reduced cost. The scan time did not affect the result indicating the potential for obtaining rapid results. The particle size of the sample affects the result, however, compromising some accuracy, larger particle sizes of the samples can also be used. The results also indicate that heterogeneous nature of the forage sample did not affect result if the samples could be ground to ≤2 mm sizes, and thus has potential for *in situ* measurements. To obtain better result drying and grinding of the sample is recommended, which would still be cheaper and quicker than wet chemical analysis. Knowing forage mineral composition will ultimately increase the efficiency of animal feeding. This work demonstrated that with the right sample preparation PXRF can obtain results comparable to digestion and ICP. Further study is warranted to extend this analysis to the samples of particle sizes >2 mm and in-the-field measurements.

## Data Availability

All datasets generated for this study are included in the manuscript and/or the supplementary files.

## Author Contributions

YS designed the experiments with TB and TG, under the supervision of LM and with technical support from BD. YS conducted the experiments, data analysis, and took the lead in writing the manuscript, which all authors have reviewed.

## Conflict of Interest Statement

The authors declare that the research was conducted in the absence of any commercial or financial relationships that could be construed as a potential conflict of interest.
